# First Description of Inheritance of a Postzygotic *OPA1* Mosaic Variant

**DOI:** 10.3390/genes13030478

**Published:** 2022-03-08

**Authors:** Svenja Alter, Navid Farassat, Sebastian Küchlin, Wolf A. Lagrèze, Judith Fischer

**Affiliations:** 1 Institute of Human Genetics, Medical Center-University of Freiburg, Faculty of Medicine, University of Freiburg, 79106 Freiburg, Germany; judith.fischer@uniklinik-freiburg.de; 2 Eye Center, Medical Center-University of Freiburg, Faculty of Medicine, University of Freiburg, 79106 Freiburg, Germany; navid.farassat@uniklinik-freiburg.de (N.F.); sebastian.kuechlin@uniklinik-freiburg.de (S.K.); wolf.lagreze@uniklinik-freiburg.de (W.A.L.)

**Keywords:** ADOA, *OPA1*, optic atrophy, postzygotic mosaic

## Abstract

Optic atrophy 1 (MIM #165500) is caused by pathogenic variants in the gene *OPA1* (*OPA1 MITOCHONDRIAL DYNAMIN-LIKE GTPase*, MIM *605290) and is inherited in an autosomal dominant manner. We describe a 6-year-old male patient with severe early onset manifestation of optic atrophy, whose parents are subjectively asymptomatic. *OPA1*-sequence analysis revealed the heterozygous missense variant NM_015560.3:c.806C>T, p.(Ser269Phe) in the patient. Segregation analysis of the parents showed that the mother carried a low-grade postzygotic mosaic of this variant, which apparently also involves germline cells. In line with this, ophthalmological investigation of the mother showed subclinical manifestation of optic atrophy 1. This is the first report of an *OPA1* postzygotic mosaic that was inherited to offspring.

## 1. Introduction

With a prevalence between 1:12,000 and 1:50,000, autosomal dominant optic atrophy (ADOA) is the most common hereditary optic neuropathy next to Leber hereditary optic neuropathy (LHON), which is primarily caused by pathogenic variants in mitochondrial DNA [1]. Several genes have been identified to cause ADOA such as *OPA3* or *DNM1L*. However, the majority of ADOA patients carry pathogenic variants in the *OPA1* gene (*OPA1 MITOCHONDRIAL DYNAMIN-LIKE GTPase*, MIM *605290). *OPA1* codes for multifunctional dynamin-like GTPase in the inner mitochondrial membrane and is essential for mitochondrial fusion. It is involved in the regulation of complex I function, mitochondrial membrane stabilization, oxidative phosphorylation and cell death pathways [2,3,4,5,6,7]. Perturbations of these pivotal functions can lead to energy deficiency, increased production of reactive oxygen species (ROS) and ultimately retinal ganglion cell death in ADOA patients [8]. Typical clinical manifestations comprise bilateral visual deterioration with a reduction in visual acuity, color vision deficits (especially along the blue–yellow axis), and central, centrocoecal or paracentral visual field defects starting in the first or second decade of life, progressing gradually. Clinical severities are heterogeneous ranging from subclinical or very mild optic neuropathy to more extensive optic atrophy with significant visual deterioration. Visual acuity usually remains better than 20/200 [9]. However, severely affected cases have been reported in larger case series [10,11,12,13].

In about 20% of cases, ADOA can manifest as the syndromic disease ADOA-Plus, which is associated with neurological signs comprising external ophthalmoplegia, proximal myopathy, ataxia, axonal polyneuropathy and sensorineural deafness [14]. Homozygous or compound heterozygous pathogenic variants in the *OPA1* gene lead to Behr syndrome, which refers to a combination of early onset optic atrophy and neurological signs such as ataxia, peripheral neuropathy and pyramidal signs [15]. There is no established therapy for ADOA. However, coenzyme Q-10 (idebenone) may slightly improve visual acuity and the visual field in affected patients [16].

*De novo* variants in *OPA1* are rare and most patients diagnosed with autosomal dominant optic atrophy 1 (MIM #165500) have an affected parent [17]. In a cohort of 980 persons with suspected hereditary optic neuropathy *OPA1*, pathogenic variants were identified in 40% of the apparently sporadic cases (157 of 392) of optic atrophy, of which only 4% (12 cases) could be confirmed to be de novo due to the lack of parental samples in the remaining cases [1]. Due to incomplete penetrance of *OPA1*-associated diseases and high inter- and intrafamilial phenotypic variability, it is also possible that a pathogenic variant can be inherited from a (seemingly) unaffected person, but the probability is low [10,13]. If a pathogenic variant cannot be detected in either parent, two scenarios are possible: (i) the index patient may have a *de novo* mutation that either occurred during gametogenesis or in early postzygotic development [18], or (ii) one of the parents carries the variant in a mosaic state, which also affects germline cells. To date, a total of 15 cases with proven *de novo* mutations in *OPA1* have been described [1,19,20], while to our knowledge, a germline mosaicism has not been reported so far [17].

Here, we present the case of a 6-year-old male patient with severe early onset manifestation of *OPA1*-dependent optic atrophy 1 and his mother with subclinical manifestation of optic atrophy 1 carrying a postzygotic *OPA1*-mosaic.

## 2. Materials and Methods

Genomic DNA was extracted from peripheral blood leucocytes, skin biopsies and oral mucosa following standard procedures. Mutation analysis was performed by next generation sequencing (NGS)-based multigene panel sequencing. Target enrichment for optic-atrophy-associated genes *OPA1* and *OPA3* was performed using a custom Agilent Haloplex or SureSelect kit (Agilent, Santa Clara, CA, USA), and subsequent sequencing was carried out on an Illumina MiSeq system (2 × 150 base pairs, paired end; Illumina, San Diego, CA, USA). Sequence data analysis was performed using the commercially available software SeqNext (JSI medical Systems, Ettenheim, Germany) and an in-house bioinformatics pipeline. The detected variant in the *OPA1* gene was validated by Sanger sequencing using standard methods and an ABI 3500 DNA Sequencer (Applied Biosystems, Foster City, CA, USA). Primer sequences for exon 8 of the *OPA1* gene (NM_015560.3, genome assembly GRCh37.p13) as well as for *MT*-*ND1*, *MT*-*ND2*, *MT*-*ND4*, *MT*-*ND4L* and *MT*-*ND6* (NC_012920.1) were established in our laboratory and can be provided upon request.

## 3. Results

A 2-year-old, otherwise healthy, boy was referred for behavioral abnormalities: in the past three months, the child’s mother had observed that he pulled objects very close for visual fixation.

Examination revealed a well-nourished male infant in no acute distress. The patient regularly approached fixation objects to a distance of about 15 cm. Binocular visual acuity was estimated as 0.3 decimal with Cardiff Cards. Pupillary light and near response were normal. Binocular alignment was unremarkable. The patient exhibited intermittent short intervals of mainly horizontal, rarely vertical, rapid conjugated eye movements. Otherwise, ocular motility was normal. Upon slit lamp examination, the anterior segment was unremarkable. Intraocular pressures (IOP) were normal. Fundoscopy revealed temporally accentuated optic pallor in both eyes. MRI of the orbit and neurocranium exhibited no abnormality, except for thinning of the optic nerves consistent with optic atrophy.

**Figure 1:**
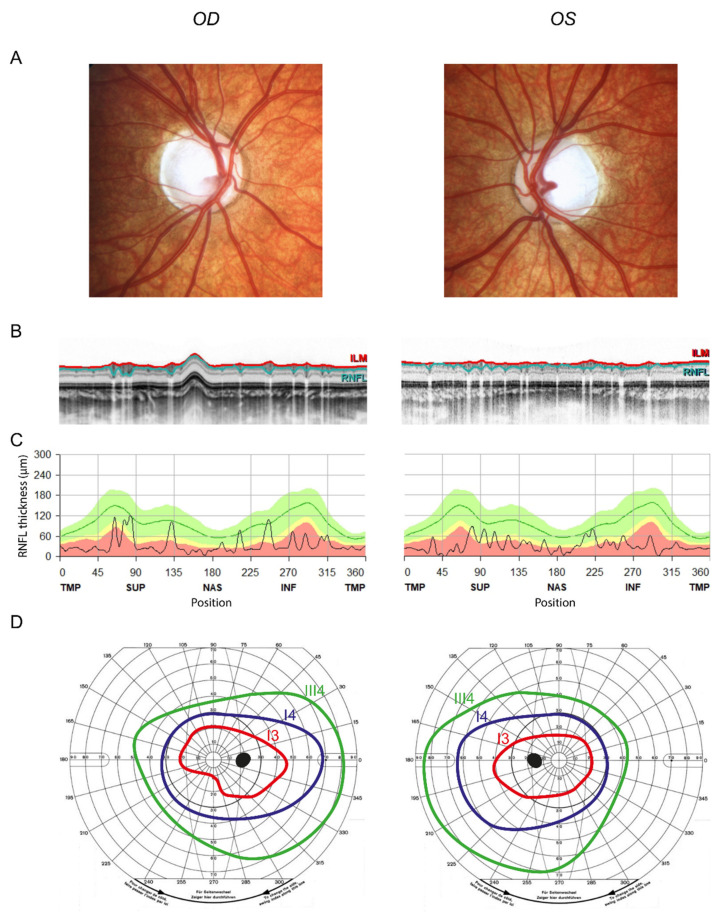
Severe optic atrophy in the son. (**A**) Fundus images showing temporally accentuated optic pallor. (**B**) Circumpapillary OCT scan (ILM in red and the border between GCL and RNFL in turquoise). Note the thinning of the RNFL. (**C**) Peripapillary RNFL thickness in all sectors, showing severe circular optic atrophy. (**D**) Goldmann perimetry showing a relatively central scotoma. Isopters: red = I3; dark blue = I4; green = III4. Note that isopter I1 was not seen. OD = right eye, OS = left eye, OCT = optical coherence tomography, ILM = internal limiting membrane, GCL = ganglion cell layer.

After four years of follow-up, the mother described that the child’s vision had deteriorated insidiously. The refractive status was emmetropia and visual acuity was 0.03 in the right eye (OD) and 0.02 in the left eye (OS). Fundoscopy confirmed temporally accentuated optic pallor in both eyes (Figure 1). Optical coherence tomography (OCT) revealed a circularly reduced peripapillary retinal nerve fiber layer (RNFL) thickness (Figure 1B,C). Goldmann perimetry showed a relative central scotoma in both eyes (Figure 1D). Examination of visually evoked potentials and quantification of the ganglion cell layer (GCL) in macular OCT scans were not possible due to patient compliance. The Ishihara color test was inconclusive due to low visual acuity. Other findings were an intermittent exotropia OS (prism test: 12°) and a high-frequency fine- to medium-beat horizontal pendular nystagmus. Of note, further history revealed that the patient had developed mild high-pitch hearing impairments in the previous six months. Otherwise, he did not complain about neurological or other problems.

**Figure 2:**
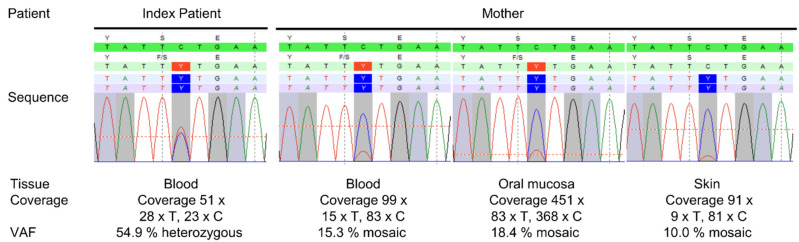
Next generation sequencing results. The variant NM_015560.3:c.806C>T, p.(Ser269Phe) in exon 8 of the *OPA1* gene was detected in a heterozygous state in DNA isolated from peripheral blood of the index patient (VAF = 54.9%). In the mother, the variant was detected with a mosaic of 15.3% in peripheral blood, 18.4% in oral mucosa and 10.0% in skin. VAF = variant allele frequency.

Using NGS gene panel analysis, we identified the heterozygous variant NM_015560.3:c.806C>T, p.(Ser269Phe) in exon 8 of the *OPA1* gene in the index patient (Figure 2). The variant has been described by Le Roux et al. (2019) [21] and was classified as likely pathogenic (denoted as c.971C>T, p.(Ser324Phe) (reference sequence NM_130837) in Additional File 1 of Le Roux et al. (2019)). In proximity to amino acid 269, further pathogenic variants causing ADOA have been described: c.803A>G, p.(Tyr268Cys) [22], c.808G>A, p.(Glu270Lys) [23], c.814C>T, p.(Leu272Phe) [24], c.815T>C, p.(Leu272Pro) [19] and c.818A>C, p.(Asp273Ala) [23]. A conserved domain or specific function has not yet been described between amino acids 268 and 273. However, the accumulation of pathogenic variants in this region indicates a functional significance for this region. Further molecular genetics analyses included a gene dosage analysis of *OPA1* using multiplex ligation-dependent probe amplification (MLPA), as well as Sanger sequencing of *MT-ND1*, *MT-ND2*, *MT*-*ND4*, *MT*-*ND4L* and *MT*-*ND6*, which include all mitochondrial mutations in LHON described to date. All mentioned analyses were normal.

We performed segregation analysis using DNA extracted from peripheral blood of the parents of the index patient via Sanger sequencing (Supplemental Figure S1). The father did not carry the variant, whereas in the mother we detected a mosaicism of the variant, c.806C>T, of around 10%. To precisely determine the degree of mosaicism, we compared DNA of the mother from tissues from different origins by next-generation sequencing (NGS): (i) peripheral blood, (ii) oral mucosa and (iii) skin. We detected the variant with a mosaic of 15.3% variant allele frequency (VAF) in peripheral blood, 18.4% in oral mucosa and 10.0% in skin (Figure 2).

**Figure 3:**
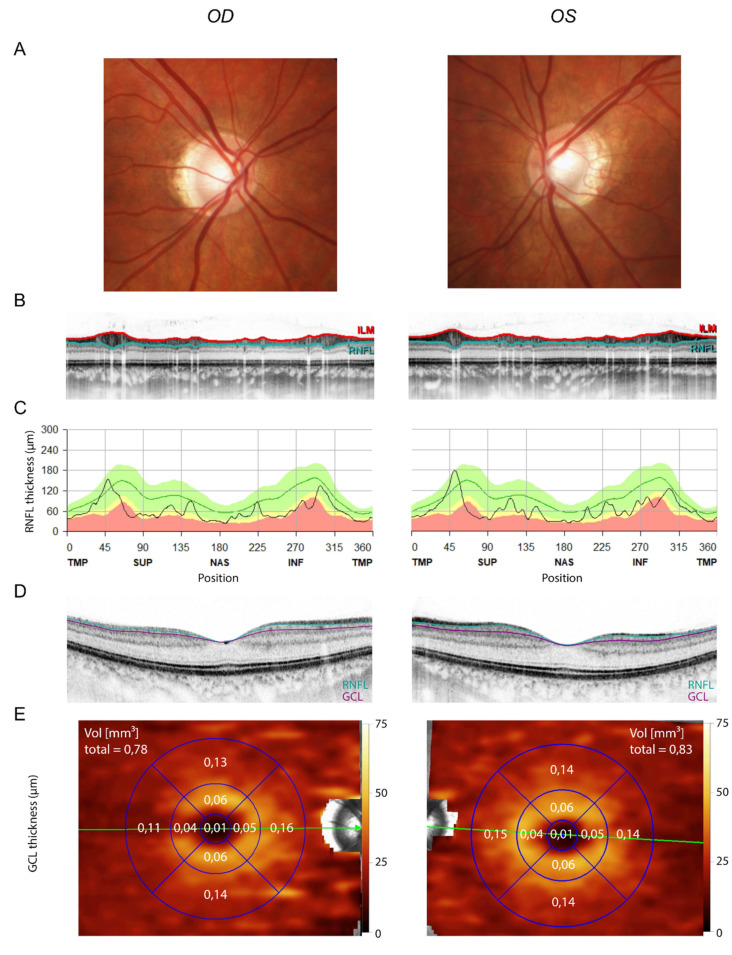
Mild optic atrophy in the mother. (**A**) Fundus images showing mild myopic papillary changes. (**B**) Circumpapillary OCT scan (ILM in red, border between GCL and RNFL in turquoise). (**C**) Macular OCT scan (border between inner nuclear layer and GCL in purple, border between GCL and RNFL in turquoise). (**D**,**E**) GCL volume quantification from macular OCT. Note the mild reduction in GCL volume (mean in the respective age group = 1.12 mm³, 5th percentile = 0.99 mm³, 95th percentile = 1.25 mm³; reference data from Meyer et al. (2021) [25]).

Due to the rather high VAF of the *OPA1* variant in the 37-year-old mother’s skin, the mother was clinically examined at follow-up to determine the clinical significance of the *OPA1* variant: best-corrected visual acuity was 0.8 decimal in both eyes (right eye: −5.25/−0.25/157°; left eye: −4.5/−0.75/50°). Fundoscopy revealed no abnormalities except a slightly tilted disc with temporal conus in both eyes (Figure 3A). OCT showed a bilateral, mild, but nearly circular reduction of the peripapillary RNFL thickness (Figure 3B,C). Likewise, her macular ganglion cell layer volume was mildly reduced to a mean of 0.78 mm³ and 0.83 mm³ on the right and left eye, respectively (Figure 3D,E; mean in the respective age group = 1.12 mm³, 5th percentile = 0.99 mm³, 95th percentile = 1.25 mm³; reference data from Meyer et al. (2021) [25]). Automated static perimetry and visually evoked potentials were unremarkable. IOPs were normal. The Ishihara test revealed no abnormalities in color perception.

Based on molecular genetics and clinical findings, we classify the variant c.806C>T, p.(Ser269Phe) in the *OPA1* gene as pathogenic based on the absence from controls (PM2, strong), the proven inheritance from a mosaic with phenotype in the mosaic carrier (PM6, strong) and multiple lines of computational evidence supporting a deleterious effect on the gene or gene product (PP3, supporting)(ACMG class 5; [26]).

## 4. Discussion

We describe the first case of a subjectively asymptomatic person carrying a postzygotic mosaic of a pathogenic *OPA1* variant involving germ cells, which was inherited to offspring. Postzygotic mosaics arise from spontaneous new mutations, mostly in very early zygotic or embryonic development [27]. The rapid advancement of NGS has led to more frequent descriptions of mosaics as the causes of neurological diseases, skin diseases and syndromic developmental disorders [28].

The two cases of *OPA1*-related ADOA presented here show highly distinct clinical presentations. The mother had subclinical optic atrophy due to a postzygotic mosaic of a pathogenic variant in the *OPA1* gene. The diagnosis of optic atrophy in the myopic mother was based on OCT measurements. Interpretation of OCTs of myopic eyes must be done with caution, as it has been shown that these measurements depend on refraction [29,30,31]. According to Savini et al. (2012), the myopic mother with a spherical refraction of −5 D was expected to have a reduction in average RNFL thickness of approximately 5% [32]. However, the mother actually exhibited a reduction in RNFL thickness of around 40% (average RNFL thickness: right eye: 61 µm; left eye: 64 µm). This cannot be attributed to myopia alone, but rather suggests that the mother had subclinical optic atrophy.

In contrast, her son had early-onset severe optic atrophy because he was a constitutional heterozygous carrier of the pathogenic *OPA1* variant. The extent of optic atrophy was severe in terms of both visual loss and age of onset. We therefore excluded relevant differential diagnoses such as glaucoma (normal IOP), cerebral mass lesion (normal MRT except for thin optic nerves), trauma (negative history) or autoimmune optic neuropathy (no subacute vision loss).

Additionally, variable intrafamilial expressivity of the phenotype might contribute to the different phenotypes of the index patient and his mother. Mosaicism and Mendelian inheritance can occur in the same genes, as it has been described, for example, for segmental neurofibromatosis type 1 and generalized neurofibromatosis type 1 [33,34]. Likewise, different phenotypic presentations have been described for pathogenic *KRT1* and *KRT10* variants, depending on whether they were present constitutively or in a mosaic state. Chia et al. (2021) describe the case of a boy with epidermolytic ichthyosis, presenting as a generalized phenotype, carrying a heterozygous pathogenic *KRT1* variant. His mother carried a postzygotic mosaic of the variant and presented with epidermolytic nevi [35]. This example shows that phenotypic manifestation correlates with the proportion of mutant cells. Based on our investigations, we assume that the low-grade mosaic in the mother is similarly causally related to the measurable abnormalities.

These observations show that *OPA1*-mosaicism can lead to mild or even subjectively asymptomatic forms of ADOA. However, if the pathogenic variant is passed on, it can cause severe manifestation of the disease in the offspring generation. This finding underlines the importance of carrier testing of apparently healthy parents of affected individuals. Adequate genetic counselling and risk assessment for further children to develop optic atrophy 1 is thus possible. The proof or exclusion of a mosaic only affecting germ cells is technically virtually impossible, especially in women. Similarly, it is difficult to prove a *de novo* mutation in the child, which occurred in early cell divisions in embryonic development. However, if a post-zygotic mosaic is detected in a parent when analyzing DNA from blood or oral mucosa, the probability for further children to be acarrier of the mutation is increased.

## Data Availability

Data are contained within the article.

## Supplementary Materials

The following are available online at https://www.mdpi.com/article/10.3390/genes13030478/s1, Figure S1: Segregation analysis: Sanger Sequencing results.
