# Supplementary figures and images for: First Description of Inheritance of a Postzygotic OPA1 Mosaic Variant
Source: Genes (Basel). 2022 Mar 8;13(3):478. doi: 10.3390/genes13030478 (PMC8948733; doi:10.3390/genes13030478)

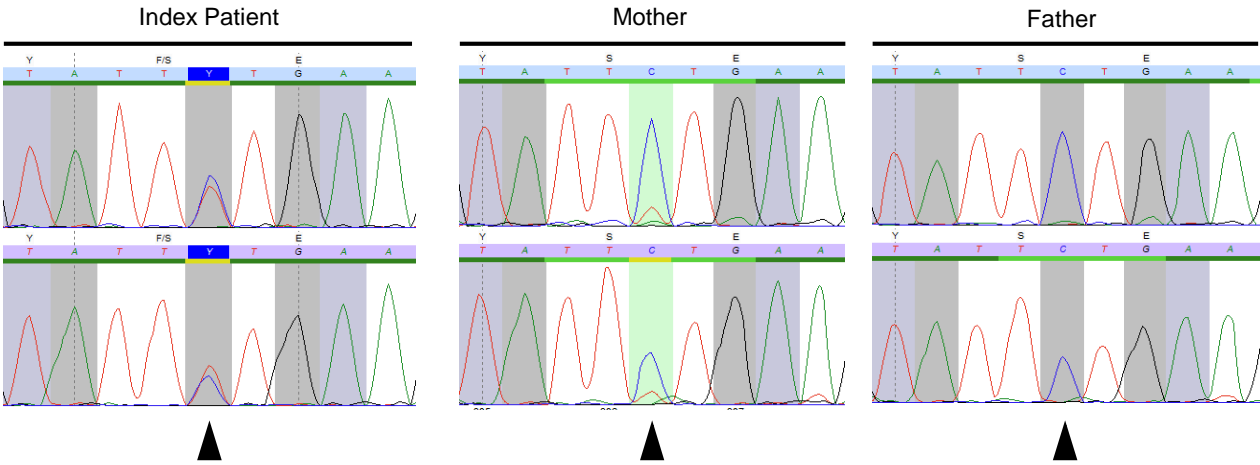

Figure S1: Segregation analysis: Sanger Sequencing results.

Supplement: Supplementary file 1 [file genes-13-00478-s001.zip › genes-1542886-supplementary.pdf]
